# Distinct interacting core taxa in co-occurrence networks enable discrimination of polymicrobial oral diseases with similar symptoms

**DOI:** 10.1038/srep30997

**Published:** 2016-08-08

**Authors:** Takahiko Shiba, Takayasu Watanabe, Hirokazu Kachi, Tatsuro Koyanagi, Noriko Maruyama, Kazunori Murase, Yasuo Takeuchi, Fumito Maruyama, Yuichi Izumi, Ichiro Nakagawa

**Affiliations:** 1 Department of Periodontology, Graduate School of Medical and Dental Sciences, Tokyo Medical and Dental University, 1-5-45, Yushima, Bunkyo-ku, Tokyo 113-8510, Japan; 2 Laboratory of Food-borne Pathogenic Microbiology, Research Center for Food Safety, Graduate School of Agricultural and Life Sciences, The University of Tokyo, 1-1-1, Yayoi, Bunkyo-ku, Tokyo 113-8657, Japan; 3 Department of Maxillofacial Surgery, Graduate School of Medical and Dental Sciences, Tokyo Medical and Dental University, 1-5-45, Yushima, Bunkyo-ku, Tokyo 113-8510, Japan; 4 Department of Microbiology, Graduate School of Medicine, Kyoto University, Yoshida-Konoe-cho, Sakyo-ku, Kyoto 606-8501, Japan

## Abstract

Polymicrobial diseases, which can be life threatening, are caused by the presence and interactions of multiple microbes. Peri-implantitis and periodontitis are representative polymicrobial diseases that show similar clinical symptoms. To establish a means of differentiating between them, we compared microbial species and functional genes *in situ* by performing metatranscriptomic analyses of peri-implantitis and periodontitis samples obtained from the same subjects (n = 12 each). Although the two diseases differed in terms of 16S rRNA-based taxonomic profiles, they showed similarities with respect to functional genes and taxonomic and virulence factor mRNA profiles. The latter—defined as microbial virulence types—differed from those of healthy periodontal sites. We also showed that networks based on co-occurrence relationships of taxonomic mRNA abundance (co-occurrence networks) were dissimilar between the two diseases. Remarkably, these networks consisted mainly of taxa with a high relative mRNA-to-rRNA ratio, with some showing significant co-occurrence defined as interacting core taxa, highlighting differences between the two groups. Thus, peri-implantitis and periodontitis have shared as well as distinct microbiological characteristics. Our findings provide insight into microbial interactions in polymicrobial diseases with unknown etiologies.

Biofilms are matrix-enclosed microbial populations that adhere to hard and soft tissue surfaces and are implicated in over 80% of known infectious diseases^1^. A technical challenge when studying the pathogenicity of polymicrobial infections by culture-dependent methods^2^ is the high prevalence of unculturable or fastidious bacteria^3^. Recent advances in molecular techniques with high-throughput sequencers have enabled the determination of polymicrobial community composition and/or interactions among microbial species in upper respiratory tract infections, osteomyelitis of the jaw, and periodontitis^4^,^5^,^6^.

Periodontitis is a representative polymicrobial biofilm-related disease that occurs in oral cavities. According to a report by the World Health Organization, disease prevalence is 15–20% in middle-aged adults (35–44 years), with 5–15% of these cases resulting in tooth loss (http://www.who.int/mediacentre/factsheets/fs318/en/). Recent evidence suggests that oral infection in periodontitis is associated with systemic diseases such as diabetes^7^ and cardiovascular diseases^8^. In addition, dental implant-based reconstruction—which has been adapted to replace conventional fixed or removable partial dentures—has led to the emergence of peri-implantitis as a serious problem in 28–56% of recipients^9^ and a major cause of implant loss. As such, effective prevention and management of peri-implantitis are essential for improving the quality of life and health of patients.

Peri-implantitis and periodontitis are polymicrobial diseases that present with similar clinical symptoms^10^. Most periodontitis cases respond favourably to treatment and show long-term stability of periodontal tissues^11^. However, clinical treatments for peri-implantitis—including those used for periodontal disease—are often ineffective^12^. In addition, peri-implantitis has been found to progress more rapidly than periodontitis in animal models^13^. To clarify the cause of differences between the two diseases, studies have characterised their respective microbiomes using culture-independent molecular approaches, including DNA hybridisation and 16S rDNA sequencing^14^,^15^,^16^. However, the causative microbial species in peri-implantitis and periodontitis are disputed. Several studies have reported the predominance of microbial species common to both diseases^16^, others unique to peri-implantitis sites have also been described^10^. One possible reason for these conflicting results is the presence of dead and/or inactive microbes in previous studies, which were based on DNA sequencing. Alternatively, microbiome samples from peri-implantitis and periodontitis sites were not obtained from the same oral cavity except in one study^15^.

To establish a means of differentiating between peri-implantitis and periodontitis, we investigated the microbial species associated with each disease as well as their functions *in situ* by carrying out a metatranscriptomic analysis at peri-implantitis and periodontitis sites in the same subjects. Our findings reveal that although they share similarities in their mRNA profiles, differences in interacting core taxa of co-occurrence networks account for the distinct etiologies of these diseases.

## Results

### Clinical characteristics of subjects and summary of sequence reads

A total of 12 patients (five men and seven women) with both peri-implantitis and periodontitis were recruited for this study. The average age was 64.5 years (range: 49–80 years); one patient was a smoker. There were no significant differences in the following clinical parameters for the two disease sites: years in function (for peri-implantitis sites only), probing depth, clinical attachment level, bleeding on probing, suppuration, and radiographic bone loss (Table 1, schematic illustrations in Supplementary Figs S1 and S2, and Supplementary Results).

### Evaluation of microbiome microbial compositions based on 16S rRNA sequences

When short metagenomic reads were analysed, there was no representative microbial composition as previously described^17^. In this approach, small subunits of the rRNA gene (16S/18S) were reconstructed using a mapping-based algorithm^17^. To assign microbial compositions with high resolution *in situ* and identify microbial species responsible for the diseases, we characterised the composition at both disease sites by sequencing total RNA and performing full- or nearly full-length reconstruction of their 16S rRNA regions^18^. The number of reconstructed 16S rRNAs (hereafter referred to as rc-rRNAs) forming operational taxonomic units (OTUs) was 58.5 ± 21.8 and 62.3 ± 20.3 in peri-implantitis and periodontitis samples, respectively (Supplementary Fig. S3a). Using the Human Oral Microbiome Database (HOMD)^19^, rc-rRNAs were assigned to 184 microbial taxa at the species level, with 150 and 164 taxa identified in peri-implantitis and periodontitis samples, respectively (Supplementary Table S2). There were no significant differences in alpha diversity, the number of OTUs (P = 0.715), or Shannon index (P = 0.834) between the two diseases (Supplementary Fig. S3a). Results from rarefaction curves indicated that a sufficient number of reads was obtained for 16S rRNA analyses (Supplementary Fig. S3b).

**Figure 1:**
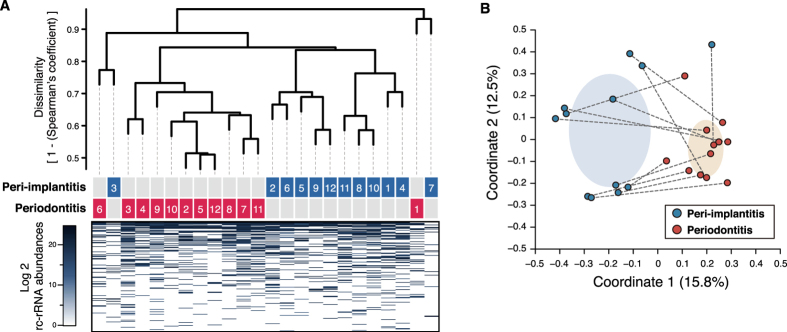
Dendrogram with a heat map and a PCoA plot of rc-rRNA profiles. (**A**) Dissimilarity values (1 − Spearman’s coefficient) were clustered using the average linkage method, as shown in the dendrogram. Disease types and patient numbers are shown under the tree. The heat map shows log2 rc-rRNA abundances for each taxon, as indicated by the colour gradient. (**B**) PCoA was carried out for the dissimilarity matrix value of 1 − Spearman’s coefficient, and 12 samples from the peri-implantitis (blue circles) and periodontitis (red circles) groups were plotted with two coordinates. The mean and standard deviation in each axis are indicated by an ellipse for each disease group. Dots corresponding to peri-implantitis and periodontitis samples from the same patient are connected by a broken line.

A dendrogram with a heat map and principal coordinates analysis (PCoA) plot (based on 1 − Spearman’s coefficient) was generated to examine differences in beta diversity. Most of the samples formed two clusters (Fig. 1), indicating differences in beta diversity between the two diseases. This was supported by an analysis of similarity (ANOSIM), which revealed that microbial compositions were dissimilar between the two groups (R = 0.399 and P = 1.00E-3). In addition, microbial composition at the genus level was diverse among samples for each disease and between both samples from each individual (Supplementary Fig. S4a). However, the predominant species were similar (Supplementary Fig. S3c and Supplementary Table S2), and no species differed significantly in terms of rc-rRNA abundance between the two diseases in Wilcoxon signed-rank tests.

### Functional profiles of microbiomes

**Figure 2:**
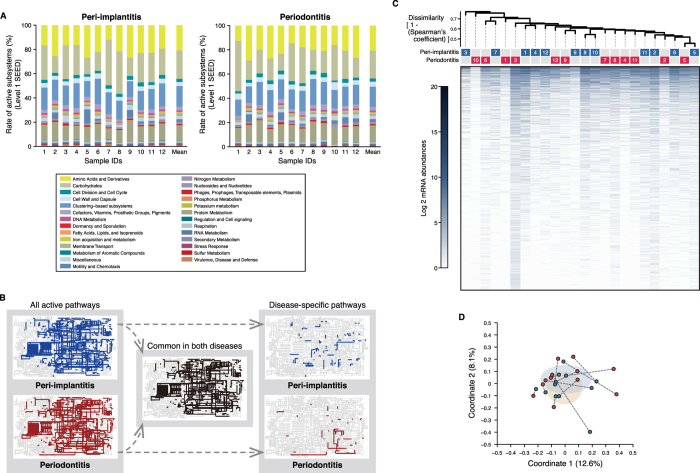
mRNA profiles obtained following assignment with SEED subsystems and KEGG and NCBI nr databases. (**A**) Percentage composition of level-1 SEED subsystems are shown for each sample ID, with the corresponding colours shown in the box below. (**B**) Active KEGG pathways present in any of the 12 samples for each disease (left map) and common and disease-specific pathways (middle and right maps, respectively). (**C**) Dendrogram constructed based on mRNA abundances during assignments with the NCBI nr database, as described in Fig. 1A. (**D**) PCoA plot prepared from mRNA profiles assigned with the NCBI nr database, as described in Fig. 1B.

The above analyses revealed dissimilarities in microbial composition between the two diseases; however, examining mRNA profiles can provide greater insight into functional differences between microbiomes^20^,^21^. We used the publically available Metagenomics Rapid Annotation using Subsystem Technology (MG-RAST) analysis pipeline to characterise putative mRNA reads in our data. We also used the SEED subsystems database to categorise functional genes into four hierarchical subsystems^22^. A total of 2461 and 2379 functional genes were assigned to peri-implantitis and periodontitis samples, respectively (Supplementary Table S3). Of these, 2006 were common to both diseases. There was no significant difference in the number of functional genes between the two diseases (P = 0.41). Among level-1 SEED subsystems, ‘carbohydrates’ was predominant at both peri-implantitis (22.1 ± 4.9%) and periodontitis (21.7 ± 5.1%) sites, followed by ‘protein metabolism’ and ‘clustering-based subsystems’ (Fig. 2A). The composition of level-1 subsystems was similar among samples of each disease and between both samples from each individual (Fig. 2A). An ANOSIM showed similarities in the functional profiles (R = 5.26E-5 and P = 0.403), and there were no genes with significantly different mRNA abundance between the two diseases in Wilcoxon tests.

Similar functional compositions were observed in the MG-RAST-processed data using the Kyoto Encyclopedia of Genes and Genomes (KEGG) database, in which metabolic pathways are listed hierarchically^23^. According to this analysis, 1380 and 1291 genes were assigned to peri-implantitis and periodontitis sites, respectively (Supplementary Table S4) and 1134 genes were shared by both diseases. There was no significant difference in the number of functional genes between the two disease groups (P = 0.370), which shared most metabolic pathways (Fig. 2B and Supplementary Table S4). Although there were some disease-specific pathways (Fig. 2B), most were active in only a single sample (Supplementary Table S4). Similar functional profiles were observed in an ANOSIM (R = −1.00E-4 and P = 0.471), and the results of the Wilcoxon tests showed no differences in expression between samples of either disease, in agreement with the SEED subsystems database assignments.

We also examined whether there were similarities when using the National Center for Biotechnology Information non-redundant (NCBI nr) protein database for functional assignment^24^ (Supplementary Fig. S1). In this database, protein functions are not categorised or organised hierarchically, but are archived with descriptions of the taxonomic origins of each function. A total of 95 487 ± 47 017 and 110 868 ± 64 965 clusters^25^ were formed from the pre-processed reads (i.e., those in which low-quality bases and putative eukaryotic organisms from raw reads were removed) for the peri-implantitis and periodontitis sites, respectively; of these, 22 988 ± 16 581 and 42 831 ± 18 992, respectively, were removed as clusters derived from putative 16S rRNA reads. The remaining clusters were further analysed as mRNA clusters (72 499 ± 48 541 and 68 038 ± 54 801 in the peri-implantitis and periodontitis samples, respectively). Using the NCBI nr database, these were assigned to 22 613 and 21 187 functional genes, respectively, for a total of 30 923 distinct mRNA clusters (Supplementary Table S5). In total, 12 737 genes were common to the two diseases; hypothetical and ribosomal proteins were predominant in both. In the dendrogram and PCoA plot, samples from each disease had similar mRNA profiles for the two diseases (Fig. 2C,D). An ANOSIM revealed similarity between the two groups (R = −1.18E-2 and P = 0.627), and the Wilcoxon tests showed no differences in read abundance of mRNA clusters or mRNA abundances for any of the genes between them. These observations were cosistent even when hypothetical and ribosomal proteins were excluded (data not shown).

### Functional profiles of putative virulence factors

**Figure 3:**
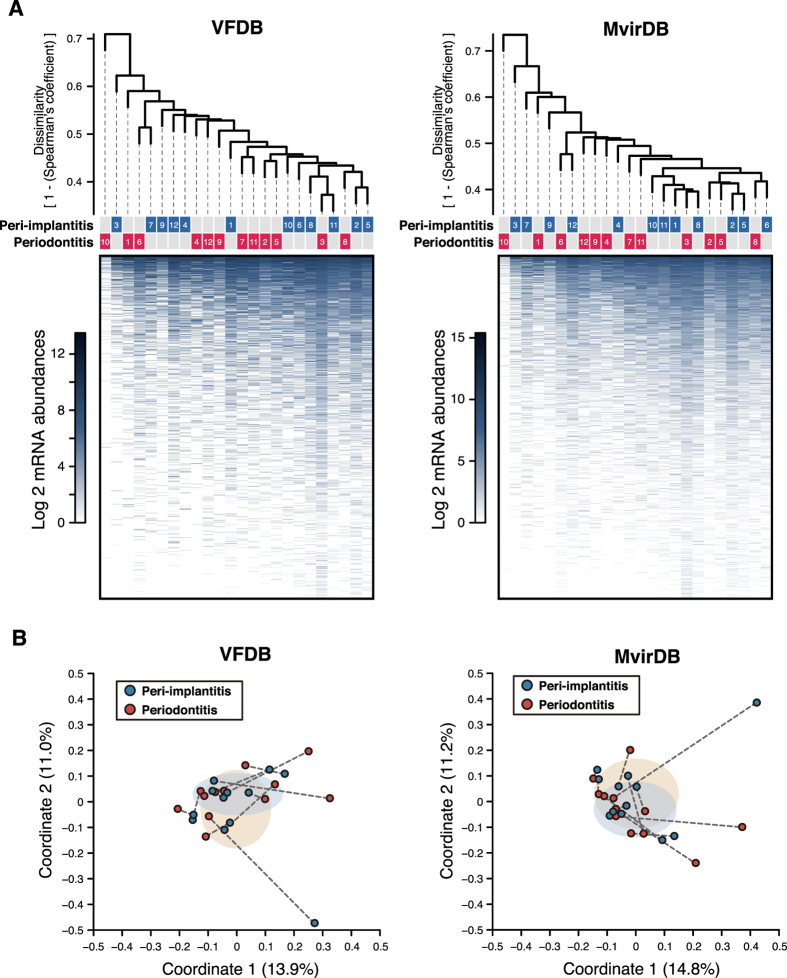
Functional mRNA profiles for assignments made with VFDB and MvirDB. (**A**) Dendrograms constructed as described in Fig. 1A. Disease types and patient numbers are shown under the tree. The heat map shows log2 mRNA abundances for each functional gene. (**B**) PCoA plots prepared as described in Fig. 1B.

The SEED subsystems, KEGG, and NCBI nr databases were used for comprehensive functional assignment of metatranscriptomic data, whereas virulence factor databases were more informative for generating detailed profiles of virulence genes^20^,^21^. Functional assignments for mRNA clusters relied on the Virulence Factors of Pathogenic Bacteria (VFDB; http://www.mgc.ac.cn/VFs/) and MvirDB (http://mvirdb.llnl.gov/) databases^26^,^27^. A total of 1579 and 1537 virulence genes in the VFDB were used to assign mRNA clusters found in peri-implantitis and periodontitis samples, respectively (Supplementary Table S6); of 1827 analysed genes, 1289 were common to both diseases. An elongation factor-encoding gene was most prevalent in peri-implantitis and periodontitis (9.89 ± 1.07% and 10.13 ± 2.26%, respectively), followed by glyceraldehyde 3-phosphate dehydrogenase, alkyl hydroperoxide reductase (*ahpC*), and enolase genes. The mRNA profiles of the two diseases were similar based on dendrograms and PCoA plots (Fig. 3A,B), which was supported by an ANOSIM (R = 3.16E-04 and P = 0.482). Wilcoxon tests revealed no differences in mRNA abundance of any virulence genes between the two diseases.

The MvirDB returned 2722 and 2622 virulence genes for the assignment of mRNA clusters in the peri-implantitis and periodontitis samples, respectively (Supplementary Table S7); 2136 genes were shared by both diseases. The predominant genes in terms of mRNA abundance were similar between peri-implantitis and periodontitis, although their rank order differed (Supplementary Table S7). As with assignments made with the VFDB, the similarity in mRNA profiles was apparent in the dendrogram-PCoA plot (Fig. 3A,B) and by ANOSIM (R = −1.82E-2 and P = 0.738), and there was no difference in the mRNA abundance of any gene between the two diseases.

**Figure 4:**
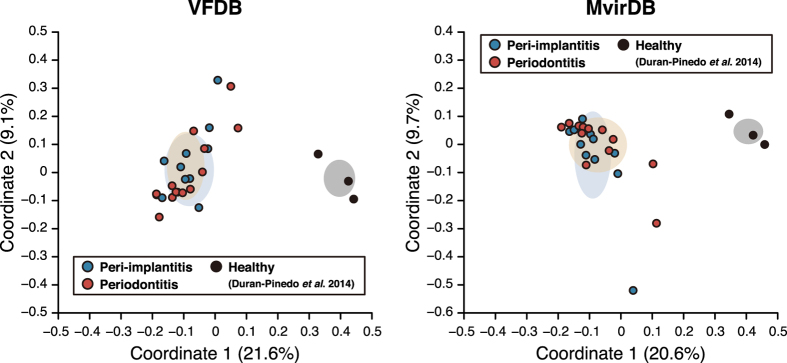
PCoA plots of functional profiles for diseased and healthy sites. PCoA plots of functional profiles for our sequence data, as well as downloaded data pertaining to healthy sites assigned with the VFDB and MvirDB, were prepared as described in Fig. 1B.

Our metatranscriptomic data were further characterised according to mRNA profiles of virulence factors, which were designated as microbial virulence (MV) types. The MV types of peri-implantitis and periodontitis sites were similar, suggesting that the two diseases were associated with similar virulence factors. This raised the question of whether MV types of diseased and healthy sites differ. MV types were assigned to RNA sequencing (RNA-seq) data from samples of healthy periodontal sites^20^, which were then compared to our data. The MV types observed in our data were distinct from those of healthy sites, as shown by PCoA plots based on assignments made with the VFDB and MvirDB (Fig. 4 and Supplementary Table S8). This was supported by ANOSIMs: we determined correlations of R = 0.980, P = 3.00E-3 and R = 0.845, P = 3.00E-3 for peri-implantitis and periodontitis, respectively, vs. healthy sites using VFDB-based profiles; and R = 0.867, P = 3.00E-3 and R = 0.783, P = 5.00E-3, respectively using MvirDB-based profiles. These results indicate that MV types of the disease sites differed from those of healthy body sites, despite similarities between the two diseases.

### Characterisation of taxonomic mRNA origins and detection of viable taxa with high mRNA abundance

**Figure 5:**
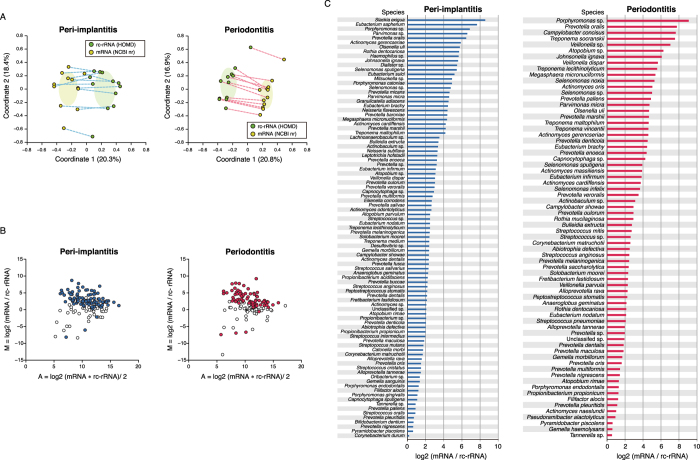
Differences in rc-rRNA and taxonomic mRNA profiles. (**A**) PCoA was performed to analyse rc-rRNA and mRNA abundances, and 12 samples in both the peri-implantitis and periodontitis groups are plotted with two coordinates. The mean and standard deviation in each axis are indicated by an ellipse. Dots corresponding to rc-rRNA and mRNA in the same patient are connected by a broken line. (**B**) VTiFs shown in MA plots for peri-implantitis and periodontitis samples. The y axis shows difference values [*M*] of rc-rRNA and mRNA abundances and the x axis shows mean values [*A*]. Coloured points indicate taxa with statistically significant differences in abundance between rc-rRNAs and mRNAs. (**C**) mRNA/rc-rRNA abundance ratio calculated for each VTiF shown in (**B**); predominant taxa (based on mean log2 ratios) for the 12 samples are shown in descending order.

A previous metatranscriptomic study reported that microbial composition determined based on 16S rRNA sequences differed from that based on taxonomic mRNA profiles in active sludge^28^. Using data that was functionally assigned with NCBI nr, we first assessed the taxonomic origin of each gene for taxonomic assignment of mRNA clusters (see Supplementary Figs S4B and S5A, Supplementary Table S9, and Supplementary Results). PCoA plots and ANOSIMs showed differences in the species compositions of mRNA and read abundances for rc-rRNAs (Fig. 5A; R = 0.496, P = 1.00E-3 in peri-implantitis and R = 0.588, P = 1.00E-3 in periodontitis samples). Taxa detected in both rc-rRNA and mRNA profiles were defined as viable taxa with *in situ* function (VTiF), of which 146 and 133 were identified in the peri-implantitis and periodontitis samples, respectively. Wilcoxon test results revealed significant differences in read abundance of each VTiF between rc-rRNA and mRNA clusters (96 and 74 taxa in the peri-implantitis and periodontitis samples, respectively; Supplementary Table S10). Log ratio–mean average (MA)-plots showed that these VTiFs mainly included taxa with high relative mRNA-to-rRNA ratio (referred to as active taxa), with mRNA abundances higher than rc-rRNA abundances (93/96 and 70/74 in peri-implantitis and periodontitis samples, respectively; Fig. 5B and Supplementary Table S10). Relative mRNA-to-rRNA ratios were indicators of the viability and functionality of microbial taxa responsible for disease etiology^29^,^30^ in the same way that the 16S rRNA-to-16S rDNA ratio has been used as an indicator of current bacterial activity^31^. In peri-implantitis samples, *Slackia exigua* and *Eubacterium saphenum* showed a relative mRNA-to-rRNA ratio >7; in periodontitis samples, *Porphyromonas* sp., *Prevotella oralis*, *Campylobacter concisus*, *Treponema socranskii*, and *Veillonella* sp. showed a relative mRNA-to-rRNA ratio >7 (Fig. 5C and Supplementary Table S10).

### VTiFs in co-occurrence networks and interacting core taxa

**Figure 6:**
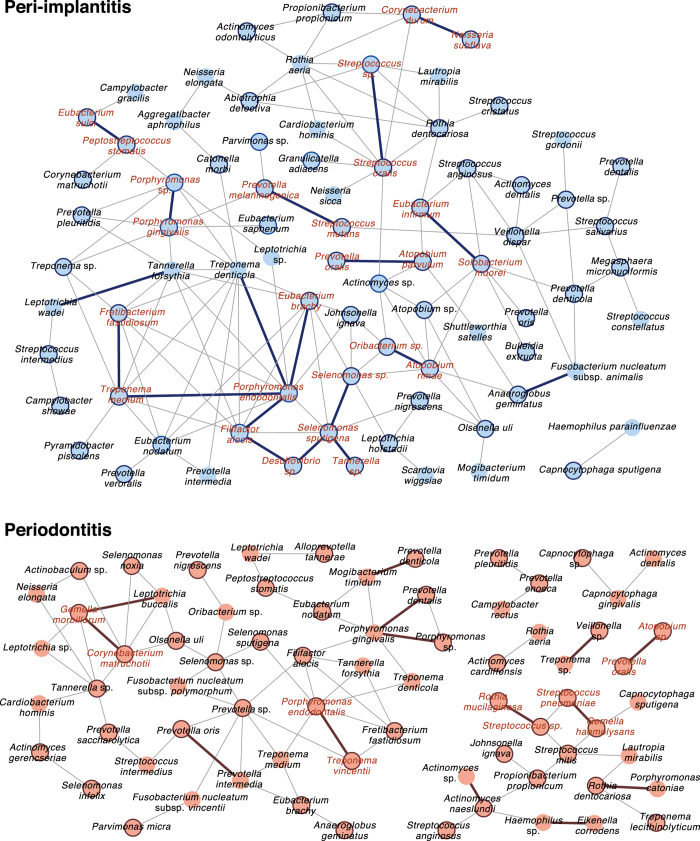
Co-occurrence networks of VTiF profiles. (**A**) All networks are shown with each microbial taxon and co-occurrence relationship indicated by a node and edge, respectively. Active taxa are indicated with bold circles, and interactions with significant co-occurrence are indicated with bold lines. Interacting core taxa are indicated in red text for peri-implantitis and periodontitis samples.

Specific microbial co-occurrence patterns in polymicrobial disease were previously characterised by correlation analysis^15^, which is a useful tool for identifying representative and important microbial associations in polymicrobial disease^32^. We analysed co-occurrence relationships in mRNA profiles of VTiFs by constructing network structures (referred to as co-occurrence networks) in which two co-occurring taxa were indicated by nodes and connected by a degree. There were one and two main network(s) with > three nodes in peri-implantitis and periodontitis samples, respectively. Means of 1.75 and 1.21 degrees per node connected 79 peri-implantitis and 71 periodontitis nodes, respectively, with clustering coefficients of 0.221 and 0.165, respectively, in these networks (Fig. 6). Active taxa were prevalent in the networks, with 60/79 and 45/71 such cases found in peri-implantitis and periodontitis samples, respectively (Fig. 6 and Supplementary Tables S10–S12). Most nodes connected by interactions with significant co-occurrence were active taxa and were detected in at least eight patients; these taxa were considered as interacting core taxa (Fig. 6 and Table 2).

## Discussion

Polymicrobial communities are composed of a variety of microorganisms, including bacteria, archaea, fungi, and viruses. The microbiome of oral cavities comprise ~600–1000 species of bacteria alone^33^. We previously showed by sequencing PCR-amplified 16S rRNA libraries that many microorganisms are responsible for chronic osteomyelitis of the jaw^4^ and peri-implantitis and periodontitis^15^. RNA-Seq is a powerful tool for examining the diversity of microbial species and their functional profiles since the obtained data reflect RNA abundance *in situ*^34^. We carried out metatranscriptomic analyses of peri-implantitis and periodontitis samples from the same oral cavities to minimise the effects of inter-individual differences. Using this approach, we observed microorganisms common to as well as differing between the two diseases with similar clinical symptoms. Moreover, we found similarities in functional profiles—including metabolic pathways and virulence factors—whereas interacting core taxa were dissimilar between the two diseases.

We first characterised the taxonomic profiles of microbial species in peri-implantitis and periodontitis samples by quantifying 16S rRNA instead of 16S rDNA; viable species were assigned while dead species were excluded^35^. Consistent with previous reports^15^,^20^, we detected high rc-rRNA abundances of *Porphyromonas gingivalis*, *Treponema denticola*, and *Tannerella forsythia* in both diseases (Supplementary Fig. S3C and Supplementary Table S2). These species are collectively known as the red complex and are prevalent in deep periodontal pockets and thus strongly associated with periodontitis^36^. Although microbial transmission from periodontal lesions to implant-adjacent tissues may underlie the onset of peri-implantitis^37^, samples from both diseases showed distinct microbial compositions (Fig. 1A,B), despite the similarities reported in our previous DNA-based study^15^. In addition, species common to both diseases with relative abundances of ≥0.5% observed here and in a previous study^15^ showed partial overlap (19.64% of 11/56 in this study), including *P. gingivalis, T. denticola, T. forsythia*, and *Eubacterium nodatum* (Supplemental Table S2), although both studies used the HOMD for taxonomic assignments. These differences could be due to amplicon bias^38^ and the detection of dead bacteria in DNA samples^35^.

It was previously reported that functional microbiome profiles of various healthy body sites were similar in terms of robustness, despite differences in microbial composition^39^. It is thus possible that peri-implantitis and periodontitis have distinct microbial compositions but similar functional profiles. Functional assignment of putative mRNA reads revealed that mRNA profiles were similar between the two diseases, with no functional gene differing significantly in terms of mRNA abundance between them according to assignments made with the SEED subsystems, KEGG, and NCBI nr databases (although some disease-specific genes were identified). The diseases shared some of the same SEED subsystems (such as ‘carbohydrates’ and ‘protein metabolism’) and KEGG pathways (Fig. 2 and Supplementary Tables S3 and S4) related to functions essential for microbial viability. However, microbial functional categories associated with the progression of both diseases showed low abundance. The level-1 SEED subsystem ‘fatty acids, lipids, and isoprenoids’—which is reportedly associated with periodontitis progression^40^—showed a prevalence of 2.22 and 2.43% at peri-implantitis and periodontitis sites, respectively, while the ‘virulence, disease, and defense’ subsystem showed a prevalence of 2.22 and 1.84%, respectively, at these sites. These were lower than percentages in environmental sample microbiomes^41^. We further analysed putative mRNA reads using the virulence-factor databases VFDB and MvirDB (Supplementary Tables S6, S7). Similarities were observed in the mRNA profiles of both disease sites despite being limited to virulence functions (Fig. 3A,B); these included genes encoding elongation factor Tu, glyceraldehyde 3-phosphate dehydrogenase, alkyl hydroperoxide reductase (*ahpC*), and enolase (Supplementary Table S6). There were no correlations between the mRNA abundance of virulence factors and clinical parameters (data not shown); indeed, the presence of virulence factors may be more important than their relative abundances at disease sites^42^. Correlations may have been observed if the virulence functions had been highly specific instead of broad.

Peri-implantitis and periodontitis showed similar mRNA profiles although these were of virulence functions; different functional profiles may occur at healthy periodontal sites. Functional pathways in healthy and periodontitis sites have been compared by metatranscriptomic analysis^43^. In addition, differences in mRNA abundance between healthy and periodontitis sites have been reported for each microbial taxon and for functional and taxonomic profiles of virulence factors^20^. When the functional properties were clustered in the same manner as enterotypes for enteric bacteria^44^, they highlighted the microbiological origin of both diseases as well as their contrast with healthy sites. In this study, we compared the MV types of both diseases with those of healthy sites using metatranscriptomic data from a previous study^20^, and demonstrated that the MV types of peri-implantitis and periodontitis sites were distinct from those of healthy sites (Fig. 4). In microbiomes showing dysbiosis, there was a reduction in microbial functional robustness in the healthy state following changes in MV type, which led to the diseased states. We therefore propose that MV types determined only by assignment of virulence factors can serve as a means of distinguishing between diseased and healthy states.

It was not always the case that the major species were also functionally predominant or that the taxonomic origins of functional genes were similar between two diseases with similar functional profiles. We therefore determined the taxonomic origins of functional genes using the NCBI nr database. The red complex species were predominant in terms of mRNA abundance and in the rc-rRNA profile, indicating that they are associated with both diseases. However, there were similarities in the taxonomic profile and origin of each mRNA. The taxonomic profiles of rc-rRNAs differed from those of mRNA clusters in both disease groups (Fig. 5A,B); this was consistent with previous results obtained by comparing microbial compositions between 16S rRNA and mRNA using the same database^28^. Since the results may have been affected by differences in the databases for the assignment of rc-RNA and mRNA clusters, we focused on VTiFs shared by the rc-rRNA and mRNA profiles and evaluated the functional activities of each VTiF by calculating their ratios of mRNA-to-rc-rRNA abundance. The anaerobic Gram-positive rod *S. exigua*^45^ and *E. saphenum* were highly abundant in peri-implantitis samples. *S. exigua* DNA was detected at slightly higher levels at periodontitis sites than at healthy sites, while *E. saphenum* DNA was more abundant at the former^46^. These two taxa were also more abundant at peri-implantitis sites than at healthy implant sites^47^. Taxa that were active in periodontitis samples (Fig. 5C) were also detected at periodontitis sites in a previous study^48^. Our analyses also revealed unclassified bacterial taxa whose functions and virulence are unknown but that highlight the dissimilarity between the two diseases. Our results demonstrate that metatranscriptomic analysis—including taxonomic mRNA classification—can detect active taxa in the microbiome.

We also observed that co-occurrence relationships in mRNA-based taxonomic profiles of VTiFs may be dissimilar between peri-implantitis and periodontitis when visualised by network structures (Fig. 6), although this was not apparent in the taxonomic mRNA profiles described above. Microbial network analyses can be used to characterise microbial interactions in environments such as soil and oral-cavity samples^32^,^49^. The networks were more complex in the peri-implantitis as compared to the periodontitis microbiome (Fig. 6); in the former, the red complex species *P. gingivalis*, *T. denticola*, and *T. forsythia* were associated with each other, whereas in the latter there were limited connections between *P. gingivalis* and *T. denticola*. Furthermore, active taxa were prevalent in the networks of both diseases (Fig. 6). In periodontitis samples, the interacting core taxa included *E. nodatum*, *Streptococcus pneumoniae*, and *Atopobium* sp., which are reportedly more abundant at periodontal sites than at healthy sites affected with periodontitis^21^. *P. gingivalis* and *Prevotella nigrescens* were among the interacting core taxa in peri-implantitis samples and were found to be more abundant^16^ while *Veillonella dispar* was less abundant^50^ at peri-implantitis sites than at healthy implant sites. Previous data showed that *P. gingivalis* is a major species in oral biofilm, which can lead to dysbiosis through changes in polymicrobial community composition^51^. In this study, *P. gingivalis* was included among interacting core taxa in the peri-implantitis but not in the periodontitis microbiome. Moreover, there were no significant interactions common to the two diseases (Table 2). These network dissimilarities are presumed to be associated with differences in disease etiology.

Our analytical methods provide an approach for characterising the interactions between the putative causal agents of diseases based on 16S rRNA and mRNA expression data. We also propose that it could be useful for characterising the MV types. Additional studies with a larger number of analysed samples could help identify differences in the functional profiles of the two diseases and clarify the role of interacting core taxa^52^.

In summary, we propose that interacting core taxa in co-occurrence networks offer novel insights into polymicrobial diseases with similar mRNA profiles. The observed similarities and dissimilarities could explain the common symptoms and differences in disease progression, respectively. In microbiomes with dysbiosis, we characterised MV types that were distinct from those of healthy sites and found that the breakdown of microbial functional robustness in the healthy state leads to a diseased state. Our findings provide a basis for the development of treatment approaches specific to peri-implantitis or periodontitis as well as a framework for metatranscriptomic studies of other polymicrobial diseases with unknown etiologies. This, in turn, can lead to a deeper understanding of the relationship between healthy and diseased states at various sites in the human body.

## Methods

### Overview of experimental workflow and statistical analyses

Experiments were performed as shown in the flowchart in Supplemental Fig. S1. Statistical analyses are schematically illustrated in Supplemental Fig. S2 and described in the Supplementary Methods.

### Ethical statement

This study was performed in accordance with the Ethical Guidelines for Clinical Studies (2008 Notification number 415 of the Ministry of Health, Labor, and Welfare). Ethical approval was obtained from the Ethics Committee of Tokyo Medical and Dental University (approval no. 661), and all patients provided informed consent before their participation.

### Patient selection and clinical assessment

Twelve subjects with at least one dental implant functioning for ≥1 year with peri-implantitis as well as one tooth with periodontitis were recruited for this study at the Tokyo Medical and Dental University Hospital Faculty of Dentistry from 2012 to 2013 (Table 1). The patients were healthy adults who had not received systemic antibiotics or oral anti-inflammatory agents in the 3 months prior to enrollment in the study. For all implants and teeth in each patient, probing depth (PD), clinical attachment level, bleeding on probing (BOP), and suppuration (SUP) were assessed at six sites per implant or tooth (i.e., mesiobuccal, buccal, distobuccal, mesiolingual, lingual, and distolingual sites). Bone loss surrounding each implant/tooth was examined with intra-oral periapical radiographs using Insight Dental Films (Eastman Kodak Co., Tokyo, Japan) obtained using the parallel technique, and was quantified by a single examiner as previously described^2^,^3^. Based on clinical data, one implant and one tooth exhibiting PD ≥ 4 mm, BOP and/or pus, and radiographic bone loss were selected for sample collection (Table 1).

### Procedure for obtaining Illumina sequence data

Subgingival plaque samples were obtained from the peri-implant and periodontal pockets at the deepest of six sites in each implant/tooth using sterile cotton and were dried to reduce eukaryotic DNA contamination. Ten pieces of paper were inserted into the pocket for 30 s, placed in a sterile tube, and stored at −80 °C. RNA was extracted from each sample, followed by cDNA synthesis and library preparation, and Illumina sequencing (Supplementary Methods). Sequence data were processed as described in the Supplementary Methods.

### Reconstruction and taxonomic assignment of putative 16S rRNA reads

Pre-processed, paired-read data were screened with EMIRGE^17^ to select putative reads derived from 16S rRNA genes and to form rc-rRNAs as OTUs. The rc-rRNAs were assigned by BLASTN against the HOMD^19^. Alpha and beta diversities were estimated and visualised by a dendrogram-PCoA plot and heat map. Detailed procedures are descried in Supplementary Methods.

### Functional assignment of putative mRNA reads with the MG-RAST pipeline

Pre-processed data were further processed with MG-RAST (v.3.3)^53^ to obtain mRNA profiles with the online BLAT program^54^, which were then analysed using the SEED subsystems^22^ and KEGG^23^ databases (Supplementary Methods).

### Formation of mRNA clusters and functional annotation

Clusters were formed from pre-processed data using the Cluster Database at High Identity with Tolerance program^25^, and putative 16S rRNA reads were removed (Supplementary Methods). The remaining (mRNA) clusters were presumed to be derived from mRNA reads and their abundance was calculated as the number of reads in that cluster. The mRNA clusters were assigned by BLASTX against the NCBI nr, VFDB, and MvirDB databases as described in the Supplementary Methods. A dendrogram-PCoA plot and heat map were prepared as described for rc-rRNA analyses.

### Evaluation of differences in taxonomic profiles between rc-rRNAs and mRNAs

The assignment of rc-rRNA and mRNA clusters took into account differences in sequence type (nucleic/amino acid) and sampled environments among the databases used. The taxonomic names of rc-rRNAs in the HOMD were manually adjusted to those of mRNA clusters, which were excluded if they were not in the list of adjusted names for rc-rRNAs, since the breadth of sampled environments was greater in the NCBI nr than in the HOMD. In addition, the % reads per kilobase per million mapped reads (RPKM) values obtained when assigning rc-rRNAs were converted to RPKM values (Supplementary Methods).

The Spearman’s coefficient was calculated for all pairs of each sample during the assignment of rc-rRNAs with HOMD and of mRNA clusters with NCBI nr. A dissimilarity matrix based on the value 1 − Spearman’s coefficient for both disease groups was used for the PCoA. The average and difference of paired log2 values of rc-rRNAs and mRNAs were calculated for each taxon to obtain MA plots for both sample groups^55^.

### Characterisation of VTiF co-occurrence networks

We first removed VTiFs with a relative abundance of <0.1% of the total number of VTiF reads^52^,^56^,^57^. Co-occurrence coefficients were then calculated using the SparCC program^58^ and the mRNA taxonomic abundances in each disease. Ten iterations were used to estimate the median correlation of each pairwise comparison, and the statistical significance of each correlation was calculated by bootstrapping with 500 iterations^56^. Taxon pairs with SparCC values ≥0.3 were considered as exhibiting a co-occurrence relationship with a positive correlation. Our criterion for significance testing was more stringent than the previously used value of ≥0.25^59^. Co-occurrence patterns were drawn using a network structure in which each taxon and co-occurrence was indicated by a node and edge, respectively, for all taxon pairs with a positive correlation. The networks were visualised using Cytoscape software v.2.8^60^.

## Additional Information

**Accession codes:** Illumina read data generated in this study are available in the DNA Data Bank of Japan (http://www.ddbj.nig.ac.jp/) under accession number DRA003492.

**How to cite this article**: Shiba, T. *et al.* Distinct interacting core taxa in co-occurrence networks enable discrimination of polymicrobial oral diseases with similar symptoms. *Sci. Rep.* **6**, 30997; doi: 10.1038/srep30997 (2016).

## Supplementary Material

Supplementary Information

Supplementary Dataset 1

Supplementary Dataset 2

Supplementary Dataset 3

## Figures and Tables

**Table 1:** Clinical characteristics of study subjects.

		Peri-implantitis	Periodontitis	P value (two-tailed paired t test)
Age		64.5 ± 7.0^a^		—
Gender (males/females)		5/7		—
Smoking/non-smoking		1/11		—
Sampled sites	Maxillary anterior	2	1	0.34
	Maxillary posterior	5	7	0.17
	Mandibular anterior	0	1	0.34
	Mandibular posterior	5	3	0.17
Years in function		8.6 ± 7.2^a^	—	—
PD (mm)		8.2 ± 2.9^a^	8.3 ± 3.1^a^	0.95
CAL (mm)		8.3 ± 2.8^a^	9.9 ± 3.9^a^	0.35
Number of sites with BOP		12	12	—
Number of sites with SUP		4	3	0.34
Radiographic bone loss (%)		52.4 ± 20.6^a^	60.9 ± 15.6^a^	0.29

^a^Values represent mean ± standard deviation.

BOP, bleeding on probing; CAL, clinical attachment level; PD, probing depth; SUP, suppuration.

**Table 2:** Interactions of interacting core taxa.

Specificity to disease groups	Species name	Log2 mRNA/rc-rRNA ratio	Number of samples detected	Species name	Log2 mRNA/rc-rRNA ratio	Number of samples detected	Positive correlation coefficient
Specific to peri-implantitis group	*Porphyromonas* sp.	6.863	12	*Porphyromonas gingivalis*	1.098	12	0.740
	*Porphyromonas endodontalis*	1.238	12	*Filifactor alocis*	1.207	12	0.643
	*Streptococcus* sp.	2.489	12	*Streptococcus oralis*	0.878	11	0.625
	*Porphyromonas endodontalis*	1.238	12	*Eubacterium brachy*	4.465	11	0.605
	*Selenomonas sputigena*	5.428	10	*Selenomonas* sp.	4.771	12	0.605
	*Streptococcus* sp.	2.489	12	*Selenomonas sputigena*	5.428	10	0.601
	*Treponema medium*	2.409	9	*Porphyromonas endodontalis*	1.238	12	0.526
	*Treponema medium*	2.409	9	*Fretibacterium fastidiosum*	2.154	12	0.496
	*Oribacterium* sp.	1.361	11	*Eubacterium sulci*	5.186	11	0.487
	*Solobacterium moorei*	2.421	9	*Eubacterium infirmum*	3.113	11	0.483
	*Oribacterium* sp.	1.361	11	*Atopobium rimae*	2.072	9	0.479
	*Filifactor alocis*	1.207	12	*Desulfovibrio* sp.	2.398	10	0.443
	*Neisseria subflava*	3.314	11	*Corynebacterium durum*	0.187	8	0.431
	*Prevotella oralis*	6.469	11	*Atopobium parvulum*	2.493	9	0.413
	*Streptococcus mutans*	1.760	11	*Prevotella melaninogenica*	2.460	11	0.392
	*Selenomonas sputigena*	5.428	10	*Desulfovibrio* sp.	2.398	10	0.375
Specific to periodontitis group	*Treponema vincentii*	4.598	11	*Porphyromonas endodontalis*	1.300	11	0.595
	*Streptococcus pneumoniae*	2.080	10	*Gemella haemolysans*	0.592	11	0.468
	*Gemella morbillorum*	1.696	11	*Corynebacterium matruchotii*	2.583	9	0.430
	*Streptococcus* sp.	2.615	10	*Rothia mucilaginosa*	2.928	9	0.384
	*Prevotella oralis*	7.763	11	*Atopobium* sp.	6.261	12	0.355
