# Supplementary material for: Distinct interacting core taxa in co-occurrence networks enable discrimination of polymicrobial oral diseases with similar symptoms
Source: Sci Rep. 2016 Aug 8;6:30997. doi: 10.1038/srep30997 (PMC4976368; doi:10.1038/srep30997)
Supplement: Supplementary Information [file srep30997-s1.pdf]

Distinct interacting core taxa in co-occurrence networks enable discrimination of polymicrobial oral diseases with similar symptoms

SUPPLEMENTARY INFORMATION

Takahiko Shiba[1†], Takayasu Watanabe[2†], Hirokazu Kachi[3], Tatsuro Koyanagi[1], Noriko Maruyama[1], Kazunori Murase[4], Yasuo Takeuchi[1]*, Fumito Maruyama[4]*, Yuichi Izumi[1] and Ichiro Nakagawa[4]

[1]*Department of Periodontology, Graduate School of Medical and Dental Sciences, Tokyo Medical and Dental University, 1-5-45, Yushima, Bunkyo-ku, Tokyo 113-8510, Japan*

[2]*Laboratory of Food-borne Pathogenic Microbiology, Research Center for Food Safety, Graduate School of Agricultural and Life Sciences, The University of Tokyo, 1-1-1, Yayoi, Bunkyo-ku, Tokyo 113-8657, Japan*

[3]*Department of Maxillofacial Surgery, Graduate School of Medical and Dental Sciences, Tokyo Medical and Dental University, 1-5-45, Yushima, Bunkyo-ku, Tokyo 113-8510, Japan*

[4]*Department of Microbiology, Graduate School of Medicine, Kyoto University, Yoshida-Konoe-cho, Sakyo-ku, Kyoto 606-8501, Japan*

[†]*These authors contributed equally to this work.*

***Corresponding authors:**

Yasuo Takeuchi, e-mail: takeuchi.peri@tmd.ac.jp, phone: 81-3-5803-5488, fax: 81-3-5803-0196.

Fumito Maruyama, e-mail: maruyama.fumito.5e@kyoto-u.ac.jp, phone: 81-75-753-4445, fax: 81-75-753-4445.

Supplementary Results

Summary of sequence reads

From total RNA samples obtained from the peri-implantitis and periodontitis sites we obtained 25 681 189 pre-processed metatranscriptomic reads with an average read length of 211.8 bp (Supplementary Table S1). There were no differences between the two diseases in terms of the number of raw ($P = 0.988$) and pre-processed reads ($P = 0.950$) (i.e., those with low-quality bases and putative eukaryotic sequences were removed). The latter were used for subsequent analyses (see the Methods section and schematic illustrations in Supplementary Figures S1, S2).

Characterisation of taxonomic origins of mRNA

Taxonomic assignment of mRNA clusters was based on the taxonomic origin of each gene according to data that was functionally assigned using the National Center for Biotechnology Information non-redundant (NCBI nr) database. The peri-implantitis and periodontitis samples had 2936 and 2820 taxonomic origins, respectively, for a total of 3492 distinct taxonomic origins (Supplementary Table S9); 2285 genes were common to the two diseases. A diverse species composition was observed among the samples of each disease and between the two disease sites in each individual (Supplementary Figure S5b). The predominant species in peri-implantitis and periodontitis samples were *Porphyromonas gingivalis* (4.73% ± 4.75%) and *Bacteroides massiliensis* (7.25% ± 9.32%), respectively (Supplementary Figure S5a). These were also the major species in each group. *B. massiliensis* was isolated from a blood culture of a newborn baby[1] and has not been previously implicated in periodontitis or peri-implantitis. An analysis of similarity (ANOSIM) revealed a correlation between the two groups ($R = -4.05E-2$ and $P = 0.869$), and Wilcoxon tests showed no differences in mRNA abundance of each taxon between the two diseases (Supplementary Figure S5b). Microbial species that were not causative agents of either disease such as the Gram-negative anaerobic rod *B. massiliensis* were predominant in assignments with the NCBI nr database but did not appear in reconstructed 16S (rc-rRNA) profiles (Supplementary Tables S2, S9).

Supplementary Methods

RNA extraction

RNA was extracted using the PowerMicrobiome RNA Isolation kit (MO BIO Laboratories, Carlsbad, CA, USA) according to a modified version of the manufacturer's protocol. Briefly, we incubated each sample with PM1 buffer overnight instead of performing the subsequent steps immediately afterward. Small RNA was removed by filtration using the NucleoSpin miRNA kit (Clontech, Mountain View,

CA, USA), followed by ethanol precipitation using Ethachinmate (Nippon Gene, Tokyo, Japan). To remove contaminating DNA, RNA was treated with 2 U of TURBO DNase (Ambion, Austin, TX, USA) at 37°C for 30 min followed by ethanol precipitation. Purified RNA was quantified using the Quant-iT RiboGreen RNA Assay kit (Life Technologies, Carlsbad, CA, USA), and RNA quality was evaluated by capillary electrophoresis with an Agilent 2100 Bioanalyzer (Agilent Technologies, Santa Clara, CA, USA).

cDNA synthesis, library preparation, and Illumina sequencing

Purified RNA was polyadenylated using the A-plus Poly(A) Polymerase Tailing kit (Epicentre, Madison, WI, USA) according to the manufacturer's protocol, then concentrated by ethanol precipitation using Ethachinmate. The polyadenylated RNA was reverse-transcribed into cDNA, and 15 cycles of amplification were carried out using the SMARTer Ultra Low RNA kit for the Illumina platform (Clontech)[4]. Sequencing libraries were prepared with the Nextera XT DNA Sample Preparation kit (Illumina, San Diego, CA, USA). Amplified DNA was quantified by real-time PCR on a LightCycler (Roche Diagnostics, Mannheim, Germany) with the KAPA Library Quantification kit–Illumina (KAPA Biosystems, Wilmington, MA, USA), and DNA quality was evaluated by capillary electrophoresis. Prepared samples from 12 patients (24 samples in total) were pooled, and the Illumina MiSeq platform was used to generate 250-bp paired-end reads.

Preprocessing of Illumina sequencing data

Illumina sequence reads were trimmed and filtered using Trimmomatic v.0.32 software[5] with the following parameters: ILLUMINACLIP:2:20:10, LEADING:15, TRAILING:15, SLIDINGWINDOW:4:15, and MINLEN:50. Raw reads composed of pairs of forward and reverse sequencing reads were processed with Trimmomatic to generate a mix of unfiltered paired and unpaired reads obtained by filtering one of the two reads in each pair. Sequences that were presumed to be of human origin (a total of 3 980 260 reads; 9.56% on average) were removed using DeconSeq v.0.4.3 software with default parameters[6]. Paired and unpaired reads in the processed data were divided using cmpfastq software (http://compbio.brc.iop.kcl.ac.uk/software/cmpfastq.php). Paired-end reads were processed with fastq-join using default parameters to combine each read pair[7], except in cases where subsequent analyses required a paired-end format.

Reconstruction and taxonomic assignment of putative 16S rRNA reads

Only paired reads were used for reconstruction and taxonomic assignment, since these were the only data acceptable for EMIRGE processing, which consisted of 40 time iterations of subsampling using default parameters. The abundance value was calculated for each rc-rRNA as the number of reads in

the rc-rRNA. The representative sequence of each rc-rRNA was subjected to a nucleotide similarity search against the Human Oral Microbiome Database v.13.2 using BLASTN. Hits with ≥ 98.5% identity and ≥ 90% query coverage were considered significant[8], and the species name of the best hit was used for taxonomic classification of rc-rRNAs. In EMIRGE, abundance values of all rc-rRNAs were normalised by conversion to reads per kilobase of transcript per million reads (RPKM), which was further converted into percentages.

RPKM values were used to estimate both alpha and beta diversities. Alpha diversity in each sample was estimated by calculating the number of rc-rRNAs and the Shannon index. Beta diversity, which reflects species richness and/or evenness among samples, was estimated by both rarefaction curves and Spearman's rank correlation coefficient between each pair of peri-implantitis and periodontitis samples. Rarefaction curves were drawn from abundance values before conversion into the RPKM with the rarefaction.single command in Mothur v.1.33.3[9]. We used Spearman's coefficient because neither of the RPKM values for each patient had parametric distributions. The value of 1 − Spearman's coefficient was used as an indicator of beta diversity, since Spearman's coefficient indicates correlation but not the degree of diversity. A dissimilarity matrix was obtained from all 1 − Spearman's coefficient values and used to construct a dendrogram using the average-linkage clustering method and perform a principal coordinates analysis (PCoA) in which each sample was plotted with two coordinates. A heat map was used to visualise log2 values of the RPKM.

Analysis with the Metagenomics Rapid Annotation using Subsystem Technology (MG-RAST) pipeline

We used only paired reads and submitted each read separately because MG-RAST had no option for accepting paired and unpaired reads in the same job. In the pipeline, hits with ≥ 70% identity, ≥ 50-bp alignment length, and E values ≤ 1e−10 were considered significant. We therefore used a more stringent threshold than was used previously[10]. All abundance values were normalised by conversion to a reads per million reads value. Bar plots were used to illustrate the composition of mRNA profiles by assignments made with level-1 SEED subsystems. Active pathways in the Kyoto Encyclopedia of Genes and Genomes database were visualised using iPath2[11].

Formation of mRNA clusters and functional annotation

The NCBI nr database (as of October 31, 2014) was used to assign protein functions to mRNA clusters. Representative sequences of mRNA clusters were subjected to amino acid similarity searches using BLASTX against the NCBI nr; hits with scores of ≥ 50 bit and E values ≤ 1e−10 were considered significant—which is more stringent than previously used criteria[12]—and the function of the best hit was assigned to the cluster. Abundance values of all mRNA clusters were normalised by

conversion to RPKM values. We determined the taxonomic origins of each mRNA, which were available in the descriptions for corresponding subjects in the NCBI nr database. These RPKM values were also used to determine the abundance of taxonomic origins.

We used the Virulence Factors of Pathogenic Bacteria (VFDB) (as of February 9, 2015) and MvirDB (as of October 9, 2014) databases to identify putative virulence factors. The former contained 2447 virulence factors and 34 228 related genes[13], while the latter contained 64 711 virulence factors[14]. Representative sequences of mRNA clusters were searched against these two databases using BLASTX. Hits with E values ≤ 1e−10, ≥ 30% query coverage, and ≥ 80% identity were considered significant, and the function of the best hit was used to assign the read; the thresholds used were more stringent than those previously applied[15].

Cluster formation and removal of putative 16S rRNA reads

From the preprocessed paired and unpaired read data, clusters were formed in cd-hit-est mode using the CD-HIT program with thresholds of ≥ 95% identity and ≥ 50% query coverage[16]. Representative sequences of each cluster were subjected to nucleotide similarity searches against the 5S, 16S, 18S, 23S, and 28S rRNA sequences of prokaryotes and eukaryotes in the ARB-SILVA (release 119) large and small subunit databases using BLASTN[17]. Sequences with nucleotide similarities to those in the indicated databases with a score of ≥ 50 bit were extracted as putative 16S rRNA reads[17].

Conversion of rc-rRNA RPKM values in the comparison of rc-rRNA and mRNA profiles

The following equation was used for the conversion: $([E/10^2] \times 10^6) \times (10^3/L)$, where E is the percent RPKM value and L is the nucleotide length of the sequence used for assignment of the cluster. The value E was converted from a percentage to parts per million, after which it was converted with a length ratio of 10^3 and L to obtain a value expressed per kilobase of transcript. This analysis enabled comparison of abundance values in the assignment of rc-rRNA and mRNA clusters using the same calculation, which was necessary since only percentages and not actual RPKM values were available in EMIRGE.

Comparison of virulence mRNA profiles of both disease sites with those of healthy periodontal sites

Microbial metatranscriptomic data from subgingival plaque samples obtained from healthy sites[18] (submission number 20130522) were compared to our data. Nucleotide lengths of the reads ranged from 35 to 151 bp. To normalise as much as possible the conditions for functional assignment in the two studies, we used only reads that were ≥ 100 bp, thereby excluding several files in FASTA format. For the remaining three healthy files, the following procedures were carried out to ensure the

equivalence of parameters in both datasets: removal of eukaryotic contaminants by Decontamination of Sequence Data (DeconSeq); clustering by CD-HIT; removal of putative 16S rRNA reads against the ARB-SILVA using BLASTN; functional assignment against the VFDB and MvirDB using BLASTX; and testing for similarities and dissimilarities by ANOSIM and PCoA. Subsampling after DeconSeq yielded a mean number of 1 070 050 reads in our data.

Statistical analysis

Two-tailed paired t tests were used to test for significant differences between datasets of both disease groups by comparing the following: the number of raw and original reads; the presence or absence of sampled sites (assigning values of 1 or 0, respectively, for sampled sites in the maxillary anterior, maxillary posterior, mandibular anterior, and mandibular posterior areas); five clinical parameters; two alpha diversity indices; and the proportion of mRNAs considered as virulence genes. ANOSIMs were used to evaluate the significance of dissimilarity between the disease groups by applying the dissimilarity matrix value of 1 − Spearman's coefficient. Each test provided an R value between the two disease groups (Supplemental Figure S2) within the range of −1 to 1 (typically between 0 and 1). R = 0 indicated that similarities between and within groups were the same on average, while R = 1 indicated that samples in a group were more similar to each other than to those from different group(s)[19]. In the ANOSIM, the P value was obtained from a permutation test, which was used to evaluate the statistical significance of the calculated R value. Wilcoxon's signed-rank test was used to test for significance differences in expression between the disease groups for each taxon or functional gene (Supplemental Figure S2).

In all the statistical tests, P values < 0.05 were considered as statistically significant, except for cases of multiple comparisons. For these, P values were converted to Q values using the Benjamini–Hochberg method as a measure of false discovery rate; significance levels were P < 0.05 and Q < 0.10. All analyses were performed using R v.3.1.1 software (R Foundation for Statistical Computing, Vienna, Austria).

Supplementary Figures

Supplementary Figure S1. Flowchart of data analyses performed in this study. Experimental and analytical procedures are shown from the sample-collection stage to the taxonomic and functional assignments made using public databases.

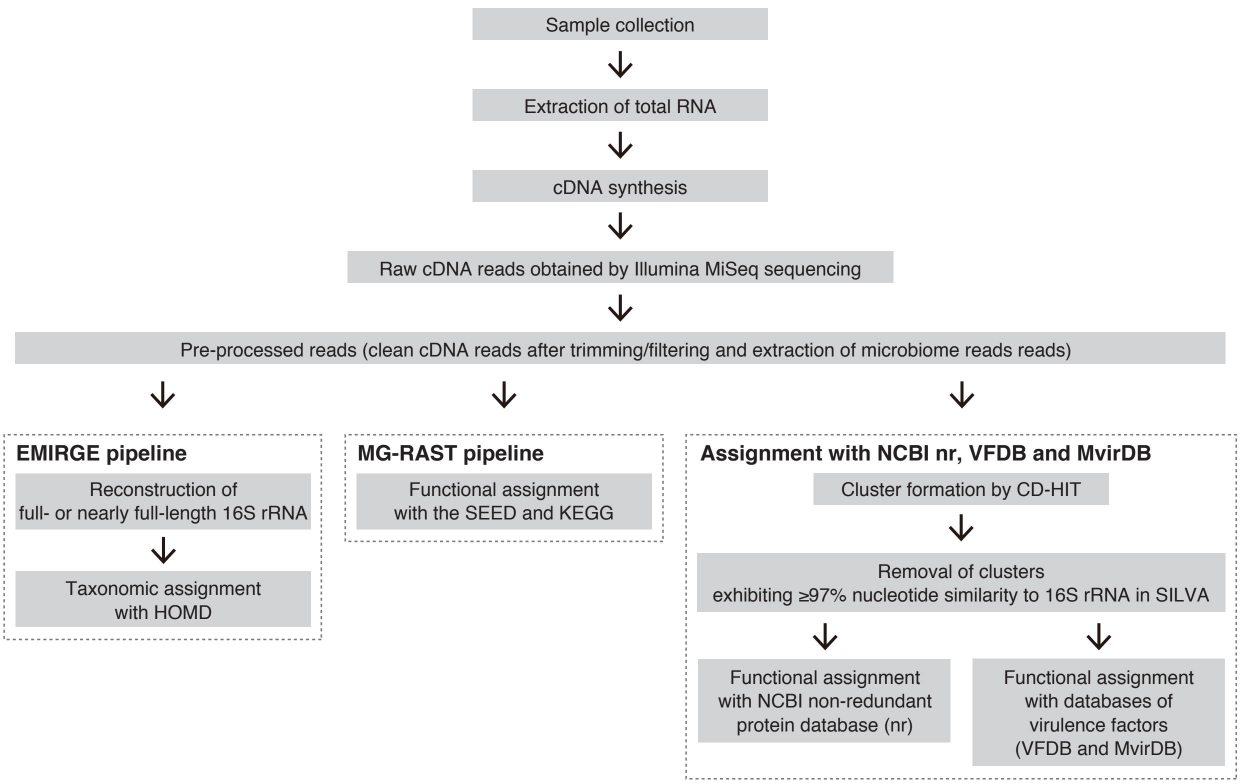

Supplementary Figure S2. Schematic overview of data analyses used for statistical analyses. Statistical tests are shown relative to tested datasets. Blue boxes indicate a value for corresponding sample and data categories. Tests with asterisks were performed only for bacterial species, the abundance profiles of which were available for both disease groups.

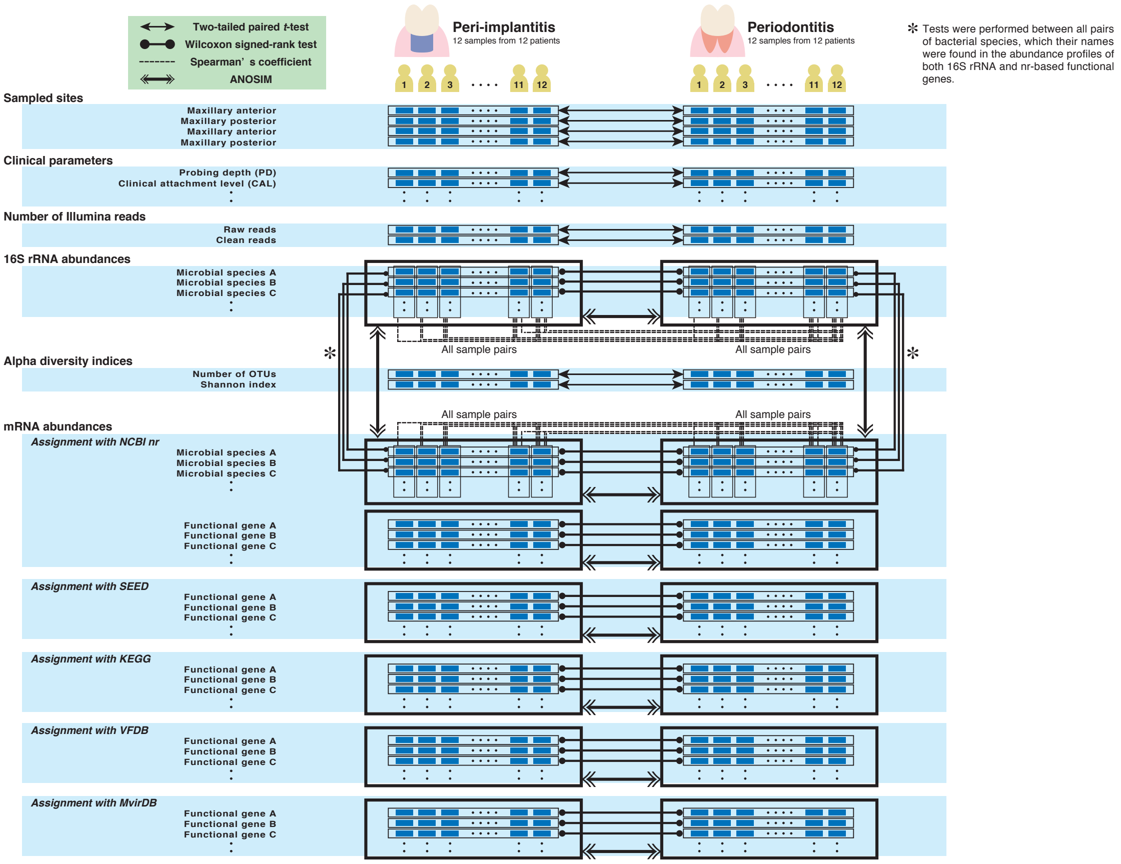

Supplementary Figure S3. Alpha diversities and relative rc-rRNA abundances of microbial species in peri-implantitis and periodontitis samples. (a) For alpha diversity measurements, the number of operational taxonomic units (OTUs) and Shannon index are shown with the mean and standard deviation. (b) Rarefaction curves were constructed from OTUs identified using EMIRGE software in the peri-implantitis and periodontitis groups. (c) Rank distributions of the taxonomic origins of rc-rRNAs in peri-implantitis and periodontitis samples. Mean rc-rRNA abundances of the 12 samples are shown in descending order with standard error bars. Disease-specific species are indicated by black dots to the right of the species name.

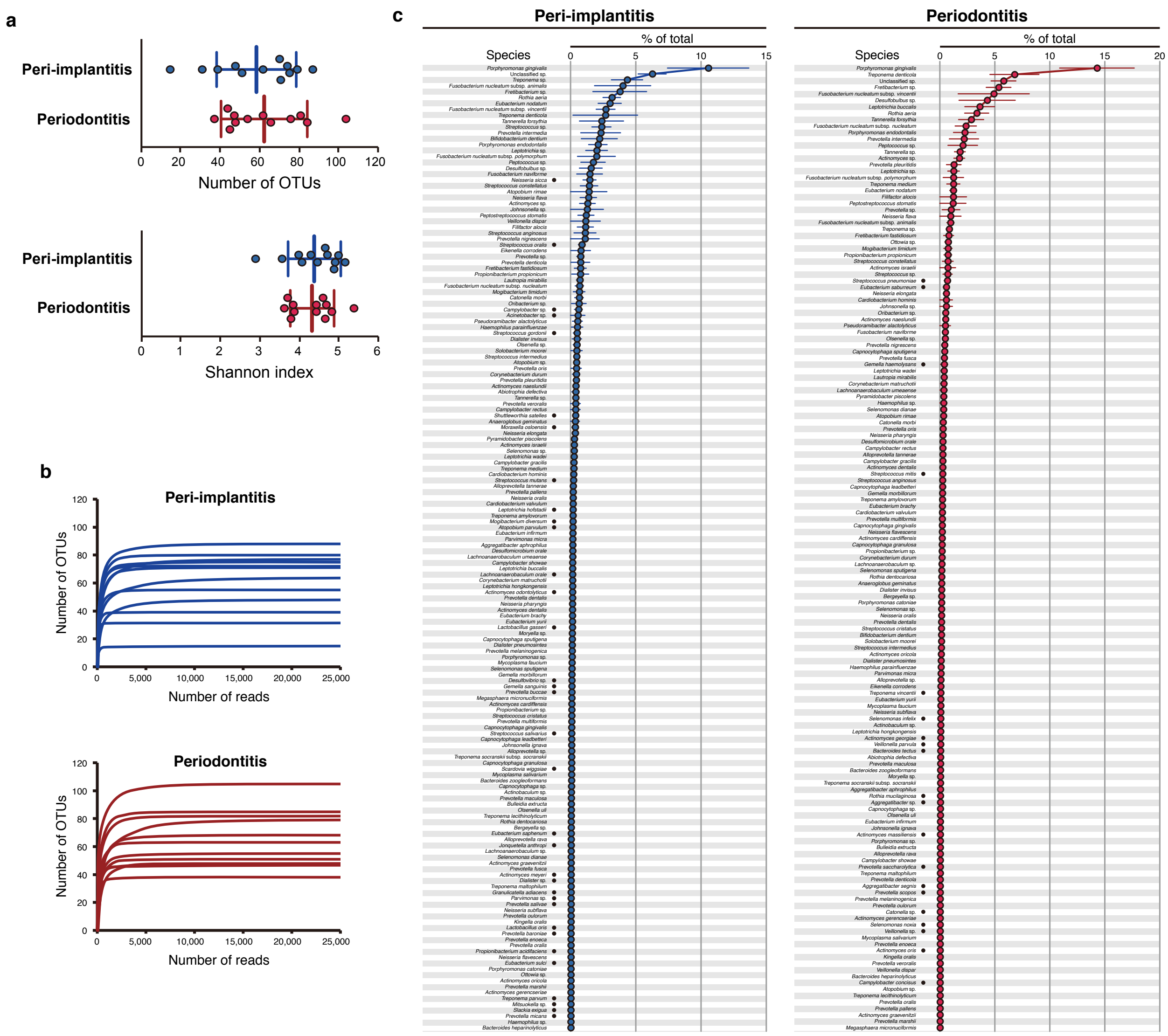

Supplementary Figure S4. Taxonomic composition of rc-rRNAs and mRNAs. (a) Percentage compositions of taxa at the rank of genus from taxonomic assignments of rc-rRNAs are shown for each sample ID, with corresponding colours indicated in the box to the right of each graph. Taxa are limited to the top 30 mean abundances observed in the 12 samples. (b) Percentage compositions of taxa at the rank of genus from taxonomic assignments of mRNAs are shown as described in (a).

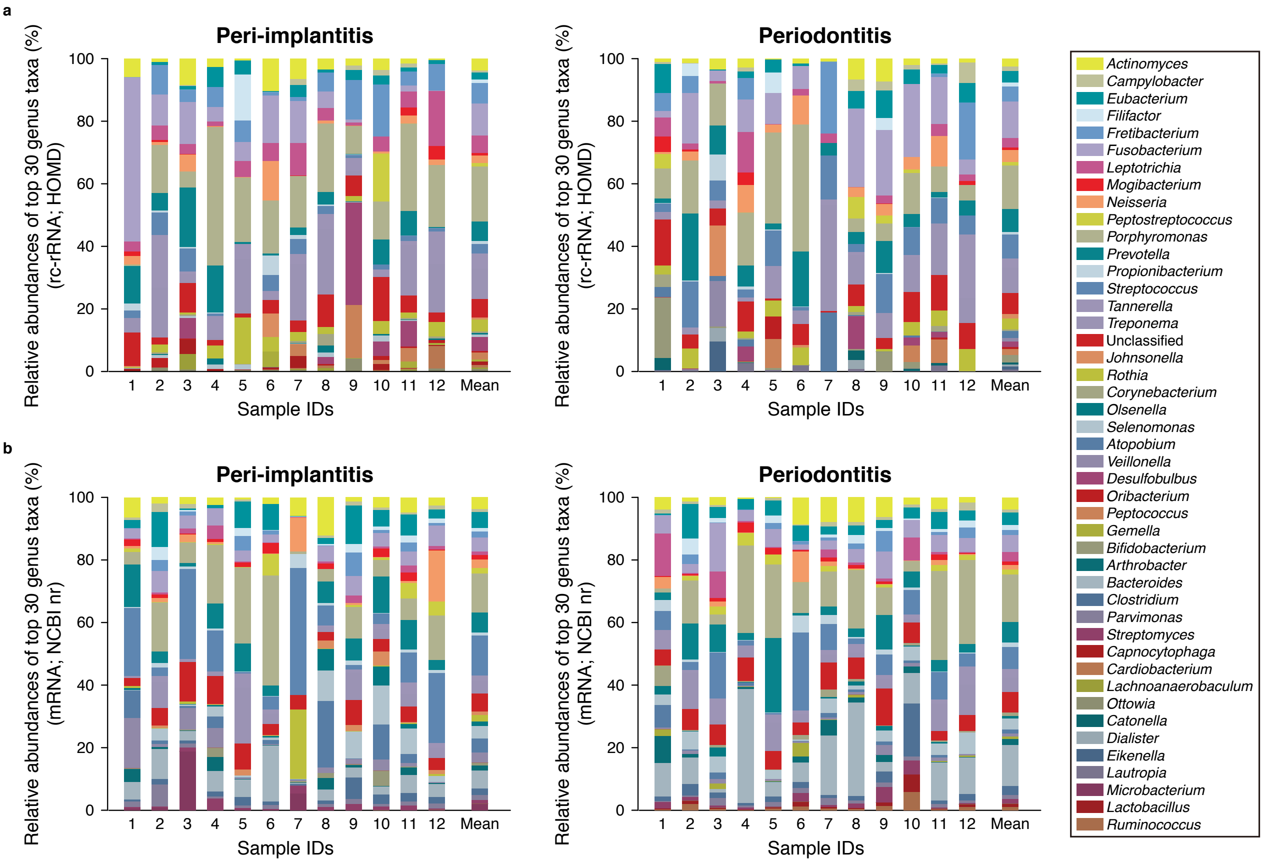

Supplementary Figure S5. Taxonomic profiles of mRNAs from assignments using the NCBI nr database. (a) Rank distributions of taxonomic origins for mRNA clusters in peri-implantitis and periodontitis samples are shown. Mean mRNA abundances of 12 samples are shown in descending order with standard error bars. Only species with ≥ 0.1% relative abundances in both diseases are shown. (b) PCoA plot prepared as described in Figure 1b.

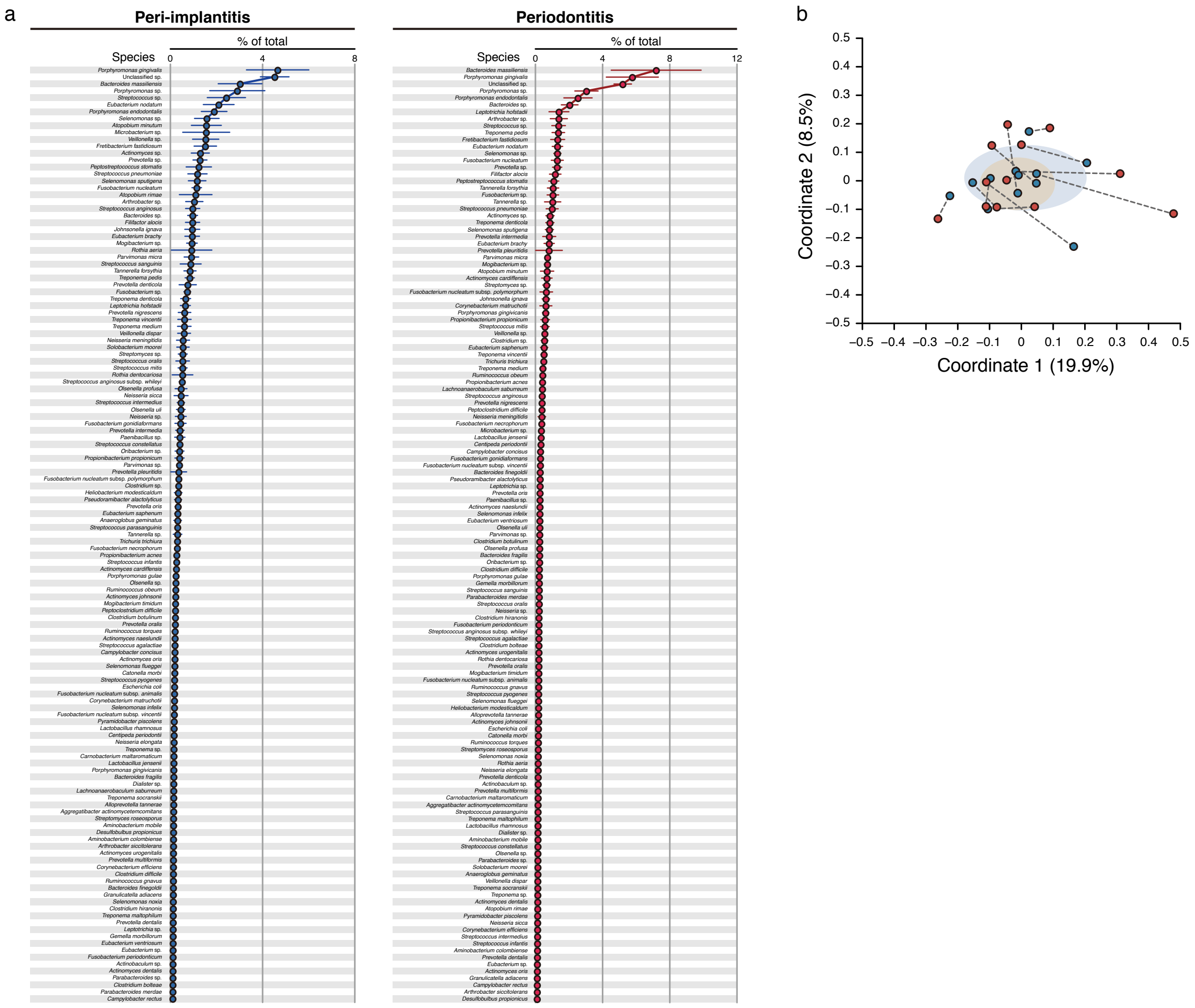

References

1 Fenner, L., Roux, V., Mallet, M. N. & Raoult, D. *Bacteroides massiliensis* sp. nov., isolated from blood culture of a newborn. *Int. J. Syst. Evol. Microbiol.* **55**, 1335-1337, doi:10.1099/ijs.0.63350-0 (2005).

2 Fransson, C. *et al.* Severity and pattern of peri-implantitis-associated bone loss. *J. Clin. Periodontol.* **37**, 442-448, doi:10.1111/j.1600-051X.2010.01537.x (2010).

3 Bahrami, B. *et al.* Effect of low fluency dentin conditioning on tensile bond strength of composite bonded to Er:YAG laser-prepared dentin: a preliminary study. *Lasers Med. Sci.* **26**, 187-191, doi:10.1007/s10103-010-0767-z (2011).

4 Ramskold, D. *et al.* Full-length mRNA-Seq from single-cell levels of RNA and individual circulating tumor cells. *Nat. Biotechnol.* **30**, 777-782, doi:10.1038/nbt.2282 (2012).

5 Bolger, A. M., Lohse, M. & Usadel, B. Trimmomatic: a flexible trimmer for Illumina sequence data. *Bioinformatics* **30**, 2114-2120, doi:10.1093/bioinformatics/btu170 (2014).

6 Schmieder, R. & Edwards, R. Fast identification and removal of sequence contamination from genomic and metagenomic datasets. *PLoS One* **6**, e17288, doi:10.1371/journal.pone.0017288 (2011).

7 Aronesty, E. Comparison of sequencing utility programs. *The Open Bioinformatics Journal* **7**, 1-8, doi:10.2174/1875036201307010001 (2013).

8 Schulze-Schweifing, K., Banerjee, A. & Wade, W. G. Comparison of bacterial culture and 16S rRNA community profiling by clonal analysis and pyrosequencing for the characterization of the dentine caries-associated microbiome. *Front Cell Infect Microbiol* **4**, 164, doi:10.3389/fcimb.2014.00164 (2014).

9 Schloss, P. D. *et al.* Introducing mothur: open-source, platform-independent, community-supported software for describing and comparing microbial communities. *Appl. Environ. Microbiol.* **75**, 7537-7541, doi:10.1128/AEM.01541-09 (2009).

10 Rajarapu, S. P., Shreve, J. T., Bhide, K. P., Thimmapuram, J. & Scharf, M. E. Metatranscriptomic profiles of Eastern subterranean termites, Reticulitermes flavipes (Kollar) fed on second generation feedstocks. *BMC Genomics* **16**, 332, doi:10.1186/s12864-015-1502-8 (2015).

11 Yamada, T., Letunic, I., Okuda, S., Kanehisa, M. & Bork, P. iPath2.0: interactive pathway explorer. *Nucleic Acids Res.* **39**, W412-415, doi:10.1093/nar/gkr313 (2011).

12 Hua, Z. S. *et al.* Ecological roles of dominant and rare prokaryotes in acid mine drainage revealed by metagenomics and metatranscriptomics. *ISME J* **9**, 1280-1294, doi:10.1038/ismej.2014.212 (2015).

13 Chen, L., Xiong, Z., Sun, L., Yang, J. & Jin, Q. VFDB 2012 update: toward the genetic diversity and molecular evolution of bacterial virulence factors. *Nucleic Acids Res.* **40**, D641-645, doi:10.1093/nar/gkr989 (2012).

14 Zhou, C. E. *et al.* MvirDB--a microbial database of protein toxins, virulence factors and antibiotic resistance genes for bio-defence applications. *Nucleic Acids Res.* **35**, D391-394, doi:10.1093/nar/gkl791 (2007).

15 Fukao, M. *et al.* Genomic analysis by deep sequencing of the probiotic *Lactobacillus brevis* KB290 harboring nine plasmids reveals genomic stability. *PLoS One* **8**, e60521, doi:10.1371/journal.pone.0060521 (2013).

16 Li, W. & Godzik, A. Cd-hit: a fast program for clustering and comparing large sets of protein or nucleotide sequences. *Bioinformatics* **22**, 1658-1659, doi:10.1093/bioinformatics/btl158 (2006).

17 Shi, Y., Tyson, G. W. & DeLong, E. F. Metatranscriptomics reveals unique microbial small RNAs in the ocean's water column. *Nature* **459**, 266-269, doi:10.1038/nature08055 (2009).

18 Duran-Pinedo, A. E. *et al.* Community-wide transcriptome of the oral microbiome in subjects with and without periodontitis. *ISME J* **8**, 1659-1672, doi:10.1038/ismej.2014.23 (2014).

19 Clarke, K. R. Non-parametric multivariate analyses of changes in community structure. *Australian Journal of Ecology* **18**, 117-143, doi:10.1111/j.1442-9993.1993.tb00438.x (1993).
